# Batch and fixed-bed column studies for selective removal of cesium ions by compressible Prussian blue/polyurethane sponge

**DOI:** 10.1039/c8ra07665k

**Published:** 2018-10-29

**Authors:** Shuquan Chang, Heliang Fu, Xian Wu, Chengcheng Liu, Zheng Li, Yaodong Dai, Haiqian Zhang

**Affiliations:** Jiangsu Engineering Laboratory of Nuclear Energy Equipment Materials, College of Material Science and Technology, Nanjing University of Aeronautics and Astronautics Nanjing 210016 P. R. China chsq@nuaa.edu.cn +86-25-52112903

## Abstract

In this work, compressible Prussian blue/polyurethane sponges (PB@PUS) for selective removal of cesium ions were prepared *via* an *in situ* radiation chemical route. The characterization results indicate that uniform PB nanoparticles were successfully synthesized and well dispersed on the porous skeleton of sponge. Batch and fixed-bed column experiments were detailedly conducted to investigate their adsorption performances. Batch adsorption experiments reveal that PB@PUS exhibited good selective removal property for cesium ions in a wide range of pH, whose maximal adsorption capacity and removal efficiency reached 68.6 mg g^−1^ and 99%, respectively. The adsorption processes could be described by the Langmuir isotherm adsorption model and pseudo-second-order adsorption kinetic model. The fixed-bed column experiments show that the breakthrough and exhaustion time obviously increased with the decrease of flow rate and initial cesium ions concentration. The breakthrough curves could be well fitted by the Thomas model and Yoon–Nelson model. The theoretical saturated adsorption capacity of PB@PUS-3 calculated from the Thomas model was 68.2 mg g^−1^. The as-prepared samples were light, stable and compressible, which can be applied in radioactive wastewater treatment.

## Abstract 2

Compressible Prussian blue/polyurethane sponges for selective removal of cesium ions were prepared and detailedly studied *via* fixed-bed column/batch adsorption experiments.

## Graphical abstract

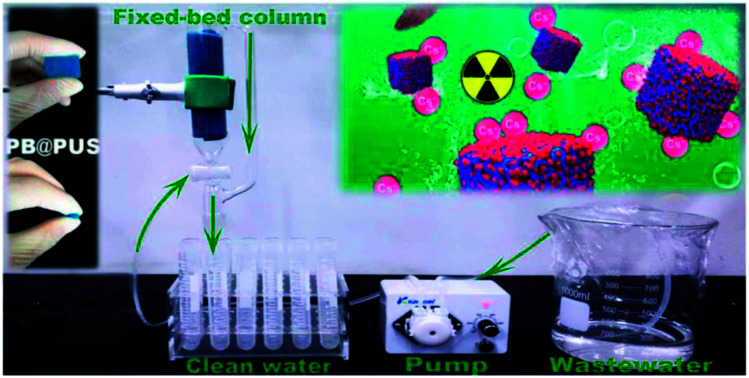

## 1. Introduction

Many kinds of radioactive wastes are produced during the application of nuclear energy and nuclear technology, which are very harmful to human beings. In the Fukushima nuclear accident, a large amount of radioactive wastewater was released into the external environment and resulted in an immeasurable ecological impact.^1,2^ Radioactive cesium-137 is the most abundant and dangerous radionuclide in radioactive wastewater, since it is one of the important fission products and has a long half-life with gamma ray emission. Also, it has extremely high solubility in water and can easily migrate in the environment like potassium.^3^ Therefore, more and more attentions have been paid to the treatment of radioactive wastewater containing cesium ions.

Many physico-chemical methods have been developed to treat radioactive wastewater, including evaporative concentration, solvent extraction, membrane separation, electrodialysis, ion exchange and adsorption *etc.*^4–6^ Among these methods, adsorption is simple, efficient and economical, which has been widely applied in radioactive wastewater treatment.^3^ A variety of adsorbents, such as zeolites, clays, permutite, activated carbon, carbon nanotubes, chitosan, alginate, cellulose and their composites, have been employed to remove cesium ions from wastewater.^7–12^ Transition metal ferrocyanides such as iron ferrocyanide (also known as Prussian blue, PB) have special lattice structure and can exchange metal ions with cesium ions.^13^ They have been widely applied in the adsorption of cesium ions from radioactive wastewater due to their low cost, non-toxicity, good stability and high selectivity.^14–19^ A commercial product CsTreat®, which is a kind of transition metal hexacyanoferrate ion exchanger, has been widely applied in nuclear power plants for radioactive cesium separation.^20,21^ The fixed-bed column is a kind of simple and efficient equipment for continuous adsorption, which has been widely used in wastewater treatment. However, traditional PB powders are difficult to be applied in fixed-bed columns due to the following reasons: (1) they are easy to be released from the columns and result in secondary contamination; (2) they cannot be completely separated from the treated solution using either filtration or centrifugation; (3) their contact area will be reduced because of the aggregation; (4) they cannot ensure the smooth flow of solution among adsorbents. In order to solve these problems, PB/GO/PVA–alginate hydrogel beads, potassium copper hexacyanoferrate/cellulose hydrogel, PB immobilized magnetic hydrogel, PB/cellulose fiber, PB/diatomite/carbon nanotubes spongiform, PB/chitosan/rayon fibers, porous three-dimensional graphene foam/PB composite, PB/PVA composite nanofiber, granulated copper hexacyanoferrate porous networks have been successfully fabricated and applied to remove cesium from wastewater.^22–32^ Modifying PB particles in bulk materials with porous structures can obviously improve their adsorption performances, which also need to be further developed according to the actual demand. In previous studies, batch adsorption experiments were usually carried out to characterize the adsorption properties of as prepared PB composites. However, their adsorption behaviour in practical application cannot be properly reflected by batch experiments.

**Fig. 1:**
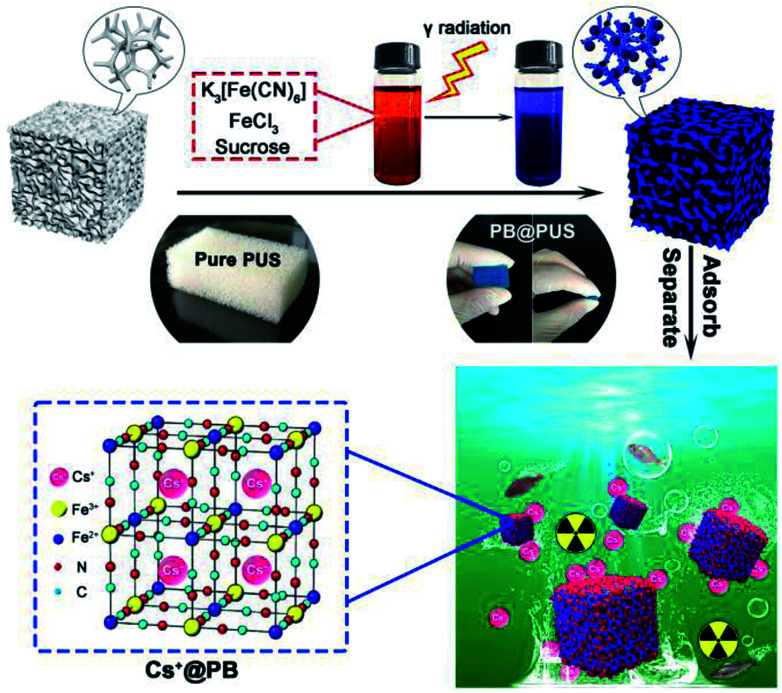
Schematic illustration for the fabrication of PB/PU sponges for cesium ions removal.

In this work, Prussian blue/polyurethane sponge complex adsorption materials for selective removal of cesium ions were proposed and prepared *via* an *in situ* radiation chemical route (Fig. 1). Their morphology and structures were characterized by scanning electron microscope (SEM), X-ray diffractometer (XRD) and Fourier transform infrared spectrometer (FT-IR). Batch experiments and fixed-bed column experiments were carried out to investigate their adsorption performances, including adsorption capacity, cesium removal efficiency, adsorption isotherms, adsorption kinetics, breakthrough curves and the influences of pH, co-existing ions, cesium ions concentration, adsorbent amount, flow rate *etc.* The fabrication strategy and adsorption mechanism were also discussed in detail. Compared with traditional PB composites, the as prepared PB/polyurethane sponge composites had obvious porous structures, high adsorption capacity and good compressibility, which could be conveniently stored, efficiently used and easily post-treated in wastewater treatment.

## 2. Materials and methods

### 2.1 Preparation of PB/PU sponges

Compressible porous polyurethane sponges with well-dispersed Prussian blue nanoparticles (PB@PUS) were rapidly prepared *via* radiation chemical route. All reagents used in this study were of analytical grade. In a typical synthesis, 0.033 g potassium hexacyanoferrate, 0.045 g iron(iii) chloride and 0.099 g sucrose were dissolved in 100 mL deionized water and mixed well; polyurethane sponges were ultrasonically cleaned in acetone and ethanol, successively; 2.1 g sponge was put in above mixed solution and bubbled with nitrogen for 1 h to remove the dissolved oxygen; above bottle was placed in the ^60^Co γ-ray equipment and irradiated for a period of time (dose rate: 0.89 kGy h^−1^; dose: 30 kGy); after irradiation, sample was washed with the deionized water to remove the unreacted reactants and free nanoparticles; the PB/PU sponge (PB@PUS-1) was obtained after vacuum drying at 60 °C for 12 h. Other two samples (PB@PUS-2 and PB@PUS-3) were prepared in the same condition except for the amount of reactants in the solution (0.133 g and 0.329 g potassium hexacyanoferrate; 0.181 g and 0.451 g iron(iii) chloride; 0.395 g and 0.987 g sucrose, respectively). Fig. 1 shows the schematic diagram of the fabrication of PB/PU sponges for cesium ions removal.

### 2.2 Characterizations of samples

Scanning electron microscope (SEM) images were taken using Hitachi S-4800 scanning electron microscope. X-ray diffraction (XRD) patterns were obtained on Bruker D8-Advance X-ray diffractometer. Fourier transform infrared (FT-IR) spectra were recorded on Bruker OPUS 80V FT-IR spectrometer.

### 2.3 Batch adsorption experiments

Because isotopes have similar chemical properties, non-radioactive cesium chloride solutions were prepared to simulate the waste water containing radioactive Cs^+^ in this study. The Cs^+^ concentration was tested using the VARIO AAS-990 atomic absorption spectrometer. The removal efficiency (*R*_e_, %) and adsorption capacity (*Q*_e_, mg g^−1^) were calculated according to the following formulas:1
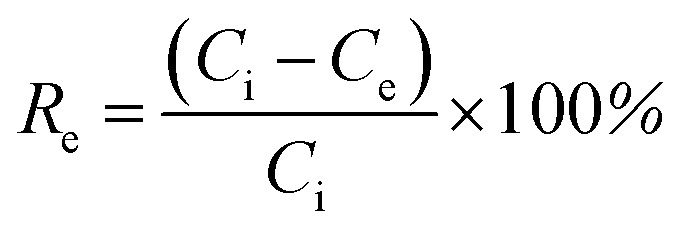
2
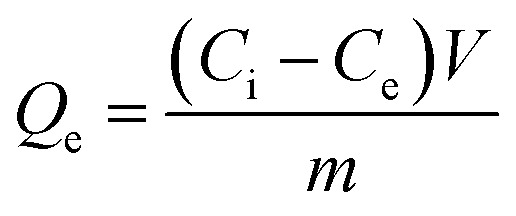
where *C*_i_ (mg L ^−1^) is the initial concentration of Cs^+^, *C*_e_ (mg L ^−1^) is the equilibrium concentration of Cs^+^, *V* (L) is the volume of the solution, and *m* (mg) is the weight of dried adsorbent.

In order to calculate their adsorption isotherms, 0.2 g adsorbent (pure PUS, PB@PUS-1, PB@PUS-2 or PB@PUS-3) was put into 25 mL Cs^+^ solution at a series of initial concentrations (5, 100, 200, 400, 600, 800, 1000, 1500, 2000 mg L^−1^) and kept for 3 h at 25 °C. The pH of solution was 7. Then, adsorbents were separated from solution using tweezers. After that, Cs^+^ concentration in the solution was tested.

In kinetics experiments, 0.2 g adsorbent was put into 25 mL 1000 mg L^−1^ Cs^+^ solution and kept for different time (5 min, 15 min, 30 min, 45 min, 1 h, 1.5 h, 2.5 h, 4 h, 5 h and 6 h) at 25 °C. After the adsorbent was separated, the Cs^+^ concentration in the solution was tested.

Different experiments were performed to investigate the influences of pH, adsorbent amount and competing ions in Cs^+^ solution. The pH of Cs^+^ solution was adjusted to 3, 5, 7, 9 and 11 with 0.1 M HCl and NaOH. The amount of adsorbents in the total volume of Cs^+^ solution was set to 2, 4, 6, 8 and 10 mg mL^−1^. In order to study their selective adsorption behaviour, adsorbents were put in the mixed solution containing Cs^+^ and competing ions (Na^+^, K^+^, Mg^2+^ or Ca^2+^) with the same concentration (1000 mg L^−1^). Unless specified otherwise, the adsorption conditions are the same as that mentioned above (Cs^+^ concentration: 1000 mg L^−1^; adsorbent amount: 8 mg mL^−1^; contact time: 3 h; temperature: 25 °C; pH: 7).

Three independent experiments were carried for each experiment. Data represent “mean ± SD (significant difference)”.

### 2.4 Fixed-bed column experiments

Fixed-bed column experiments were carried to investigate the dynamic adsorption properties of samples. Adsorbents were put in the glass column with the inner diameter of 2.6 cm. The height of adsorbents in the column was 10 cm. The Cs^+^ solution was delivered with up-flow mode using a constant-flow pump (Kamoer KCP3-X) at room temperature. The Cs^+^ concentration was 10, 20 and 30 mg L^−1^, respectively. The flow rate of solution was 3, 4 and 5 mL min^−1^. The effluent was collected from the top of the column at regular time intervals and then analyzed by atomic absorption spectrophotometer.

The loading behaviour of samples into the fixed-bed column was expressed in terms of the normalized concentration *C*_*t*_/*C*_0_ (where *C*_0_ and *C*_*t*_ are the inlet Cs^+^ concentration and outlet Cs^+^ concentration at time *t*, respectively) as a function of time (*t*) for a given bed height, giving a breakthrough curve. When the column was exhaust, the total effluent volume (*V*_eff_, mL) was calculated from the following equation:

3 *V*_eff_ = *νt*_total_

where *v* (mL min^−1^) and *t*_total_ (min) are the volumetric flow rate and the time of exhaustion, respectively.

For a given feed concentration and flow rate, the area under the breakthrough curve can be obtained by integrating the adsorbed concentration (*C*_0_ − *C*_*t*_) *versus t* (min) plot. The total adsorption amount *q*_total_ (mg) was obtained from the following equation:4
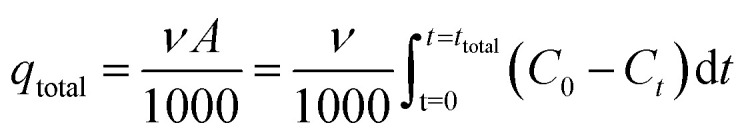


The equilibrium Cs^+^ maximum capacity (*q*_s_, mg g^−1^) of the column was calculated as the following:5
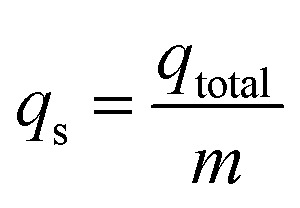
where *m* (g) is the dry weight of adsorbent in the column.

Total amount of cesium ions entering column (*m*_total_, g) was calculated from the following equation:6
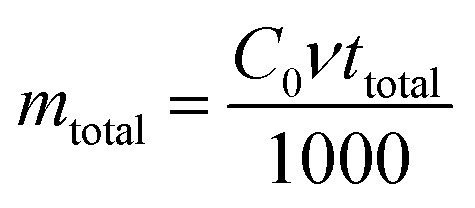


The Cs^+^ removal percentage (*R*_total_, %) for the fixed-bed column at saturation was calculated as the following:7
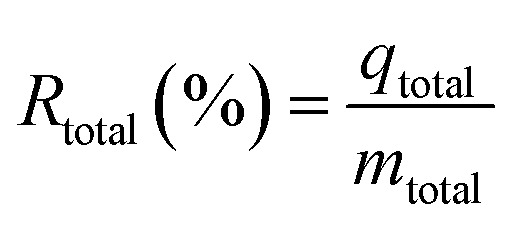


The breakthrough adsorption capacity (*q*_b_, mg g^−1^) at the time of breakthrough (*t*_b_, min) was determined using the same computational method as that of *q*_s_.

## 3. Results and discussion

### 3.1 Morphology and structure of as prepared composites

**Fig. 2:**
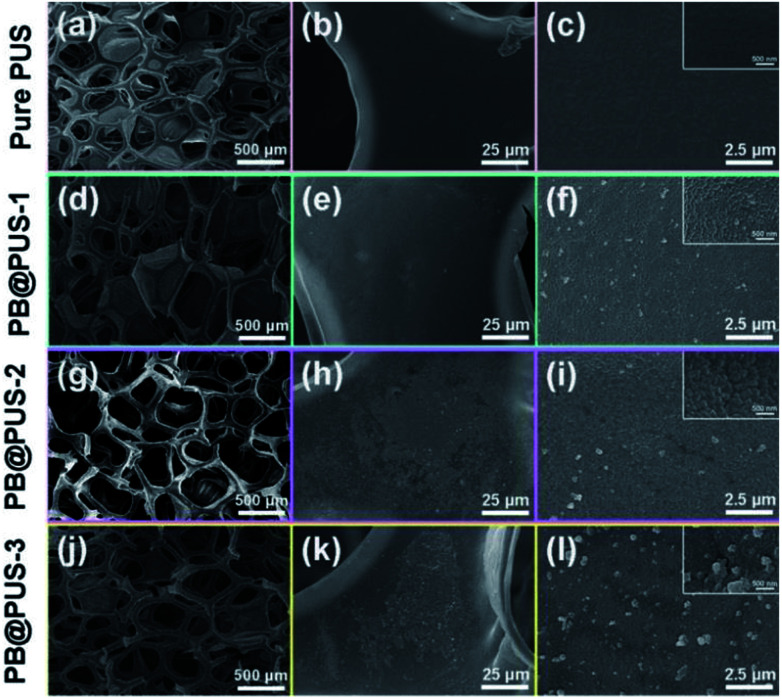
SEM images of pure PU and PB/PU sponges.

The morphology of PB/PU sponges fabricated on different conditions were investigated and shown in Fig. 2. Pure PU sponge has obvious porous structures and smooth internal surface. PB/PU sponges also have obvious porous structures but rough surfaces. In PB@PUS-1 and PB@PUS-2, 80–100 nm and 100–200 nm PB nanoparticles were well distributed on the surface of porous structures. In PB@PUS-3, irregular PB nanoparticles were densely distributed on the surface of porous skeleton. The shape, size and distribution of PB particles can be affected by the concentrations of reactant and small molecule stabilizer. It is easier to form uniform and well-distributed PB nanoparticles when the concentration of reactants is lower. As the increase of reactants concentration, more PB nanoparticles are rapidly formed and aggregate on the surface of porous skeleton. All samples have good intensity, flexibility and compressibility, which will be beneficial for adsorption and storage.

**Fig. 3:**
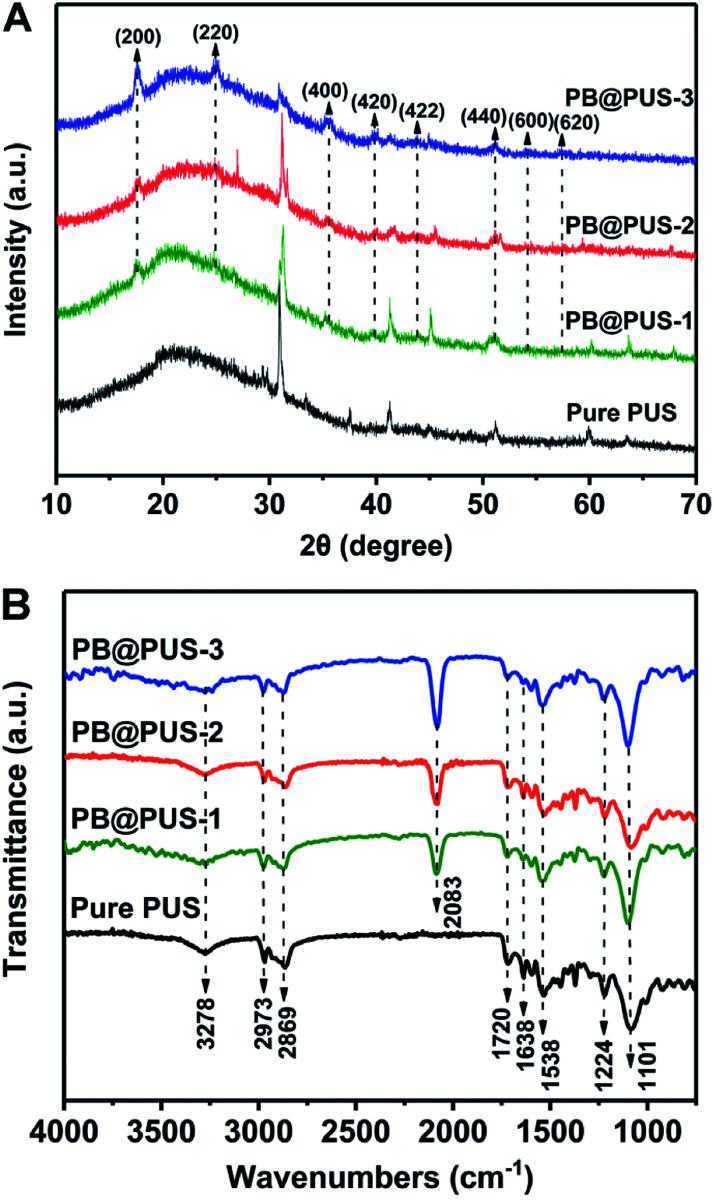
XRD patterns (A) and FT-IR spectra (B) of pure PU and PB/PU sponges.

The X-ray diffraction patterns of pure PU and PB/PU sponges fabricated on different conditions were shown in Fig. 3A. Compared with pure PU sponges, PB/PU sponges have several new diffraction peaks at around 17.5°, 24.8°, 35.4°, 39.7°, 43.8° and 51.0°, which correspond to the (200), (220), (400), (420), (422) and (440) planes of Prussian blue (JCPDS 73-0687).^33^ The FT-IR spectra of samples are shown in Fig. 3B. The peaks at around 3278 cm^−1^, 2973 and 2869 cm^−1^, 1720 cm^−1^, 1638 cm^−1^, 1538 cm^−1^, 1224 cm^−1^, 1101 cm^−1^ are separately ascribed to O–H and N–H stretching, C–H stretching absorption bands, C=O stretching, O–H bending vibration, C–H stretching vibrations, C–N stretching vibrations, C–O–C stretching vibrations.^34^ Compared with pure PU sponge, a strong peak at around 2083 cm^−1^ appears in PB/PU sponges, which is the characteristic peak of the C≡N stretching vibration in Prussian blue.^35^ XRD and FT-IR results reveal that the nanoparticles on the surface of porous skeletons are Prussian blue. They also indicate that the structures of PU sponges are not obviously damaged under irradiation.

### 3.2 Static adsorption properties

**Fig. 4:**
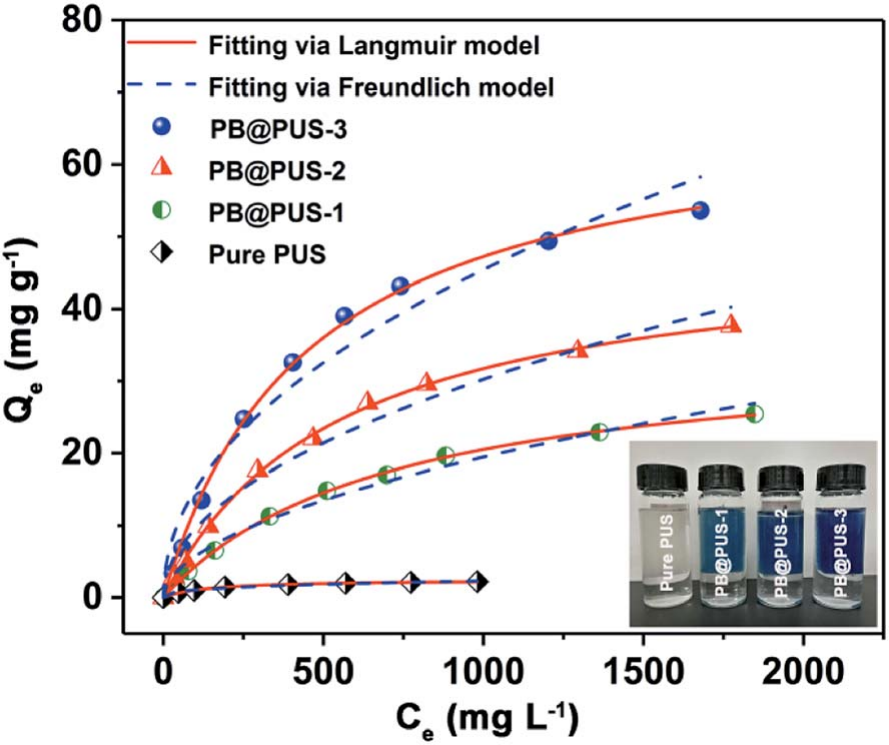
Adsorption isotherms of pure PU and PB/PU sponges.

The adsorption isotherms of samples were investigated by batch experiments. As is shown in Fig. 4, the adsorption capacities of pure PU and PB/PU sponges increase as the increase of initial Cs^+^ concentration. The adsorption isotherm can reveal the interactive behavior between the adsorbents and ions in the solution. Therefore, the adsorption data were fitted with Langmuir and Freundlich isotherm adsorption models,^36^ which are presented by the following equations:8
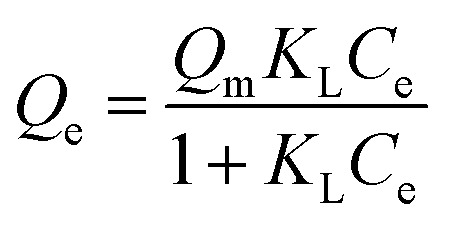

9 *Q*_e_ = *K*_f_*C*_e_^1/*n*^

where *Q*_e_ (mg g^−1^) is the adsorption capacity, *C*_e_ (mg L^−1^) is the equilibrium metal ions concentration, *Q*_m_ (mg g^−1^) is the maximum adsorption capacity, *K*_L_ (L mg^−1^) is the Langmuir constant, *K*_f_ and *n* are the Freundlich constant and linearity index, respectively.

The adsorption isotherms and parameters fitting with the Langmuir and Freundlich models are given in Fig. 4 and Table 1.

**Table 1:** Langmuir and Freundlich adsorption isotherm parameters

Adsorbent	Langmuir model			Freundlich model		
	*Q*_m_ (mg g^−1^)	*K*_L_ (L mg^−1^)	*R* ^2^	*K*_f_ (mg^1−1/*n*^ L^−1/*n*^ g^−1^)	1/*n*	*R* ^2^
Pure PUS	2.49	0.00726	0.994	0.225	0.340	0.986
PB@PUS-1	34.9	0.00143	0.999	0.508	0.528	0.984
PB@PUS-2	49.2	0.00182	0.999	0.969	0.498	0.976
PB@PUS-3	68.6	0.00221	0.998	1.64	0.481	0.965

Between the two isotherm models, the Langmuir model fits the adsorption data better than the Freundlich model, which can be confirmed by the correlation coefficients. Above results indicate that adsorption sites in two samples are uniform and belong to single layer adsorption. According to the Langmuir model, the maximal adsorption capacity of PB@PUS-3 is 68.6 mg g^−1^, which is almost twenty eight times compared to that of pure PU sponge (2.49 mg g^−1^). The excellent adsorption property of PB/PU sponge is attributed to the synergetic effects between PB and PU sponge. PB nanoparticles have special lattice and can exchange its potassium ions with cesium ions, which provide numerous specific adsorption sites for cesium ions. PU skeletons of sponge prevent the aggregation of PB nanoparticles, increase the specific surface area and provide enough channels for the solution, which increase the contact opportunity between PB and cesium ions.

**Fig. 5:**
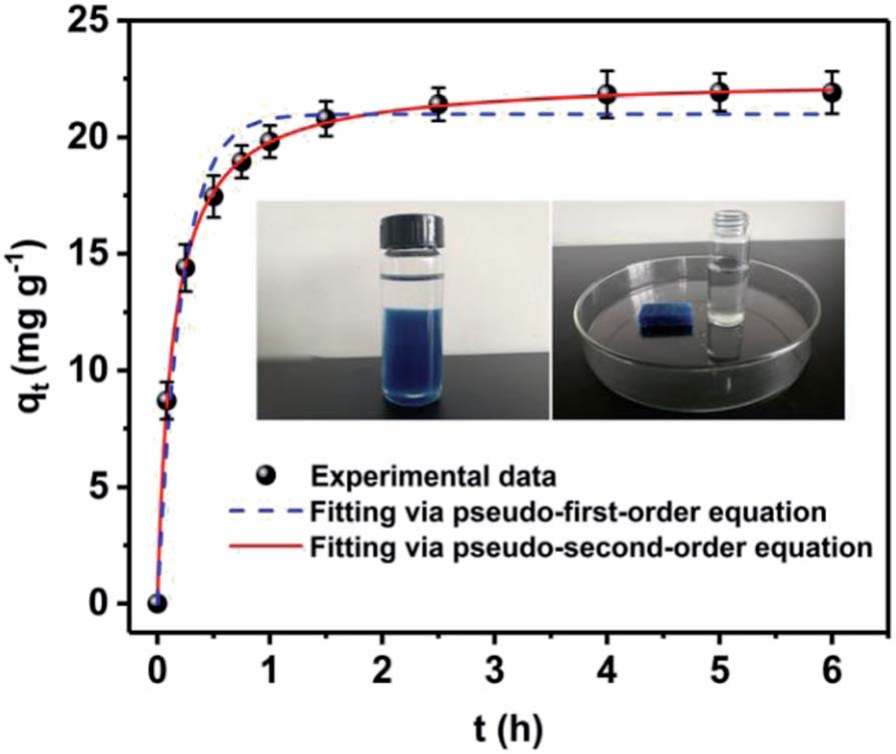
Adsorption kinetic curve of PB@PUS-2 sample. Data represent “mean ± SD” from three independent experiments.

In order to ascertain the adsorption equilibrium time for Cs^+^, the adsorption capacity of samples under different adsorption time was tested and shown in Fig. 5. The adsorption process can be divided into two stages. In the first stage, the adsorption processes increase rapidly. Its adsorption capacity can reach 17.5 mg g^−1^ within 30 min. Then, adsorption gradually increases and reaches equilibrium in 2.5 h. After that, there is no significant adsorption for Cs^+^. To describe the adsorption kinetics, the pseudo-first-order adsorption kinetic model (eqn (10)) and pseudo-second-order adsorption kinetic model (eqn (11)) were employed to fit the experimental data.

10 *q*_*t*_ = *q*_e_(1 − *e*^−*k*_1_*t*^)

11
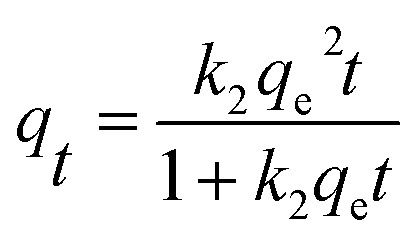
where *q*_e_ (mg g^−1^) and *q*_*t*_ (mg g^−1^) are the adsorption capacities at equilibrium and at time *t* (min), respectively; *k*_1_ (min^−1^) and *k*_2_ (g mg^−1^ min^−1^) are the adsorption rate constants of pseudo-first-order model and pseudo-second-order model, respectively.

The adsorption kinetic parameters fitting with pseudo-first-order and pseudo-second-order models are shown in Table 2 and Fig. 5. The results reveal that the pseudo-second-order model (*R*^2^ = 0.999) fits the adsorption data better than pseudo-first-order model (*R*^2^ = 0.976). Adsorption processes of PB/PU sponge might be controlled by chemical interactions, which include the ions exchange between Cs^+^ and K^+^ in PB.^37^

**Table 2:** Pseudo-first-order and pseudo-second-order kinetic parameters

Adsorbent	Pseudo-first-order equation			Pseudo-second-order equation		
	*q*_e_ (mg g^−1^)	*k*_1_ (min^−1^)	*R* ^2^	*q*_e_ (mg g^−1^)	*k*_2_ (g mg^−1^ min^−1^)	*R* ^2^
PB@PUS-2	21.0	4.62	0.976	22.5	0.322	0.999

**Fig. 6:**
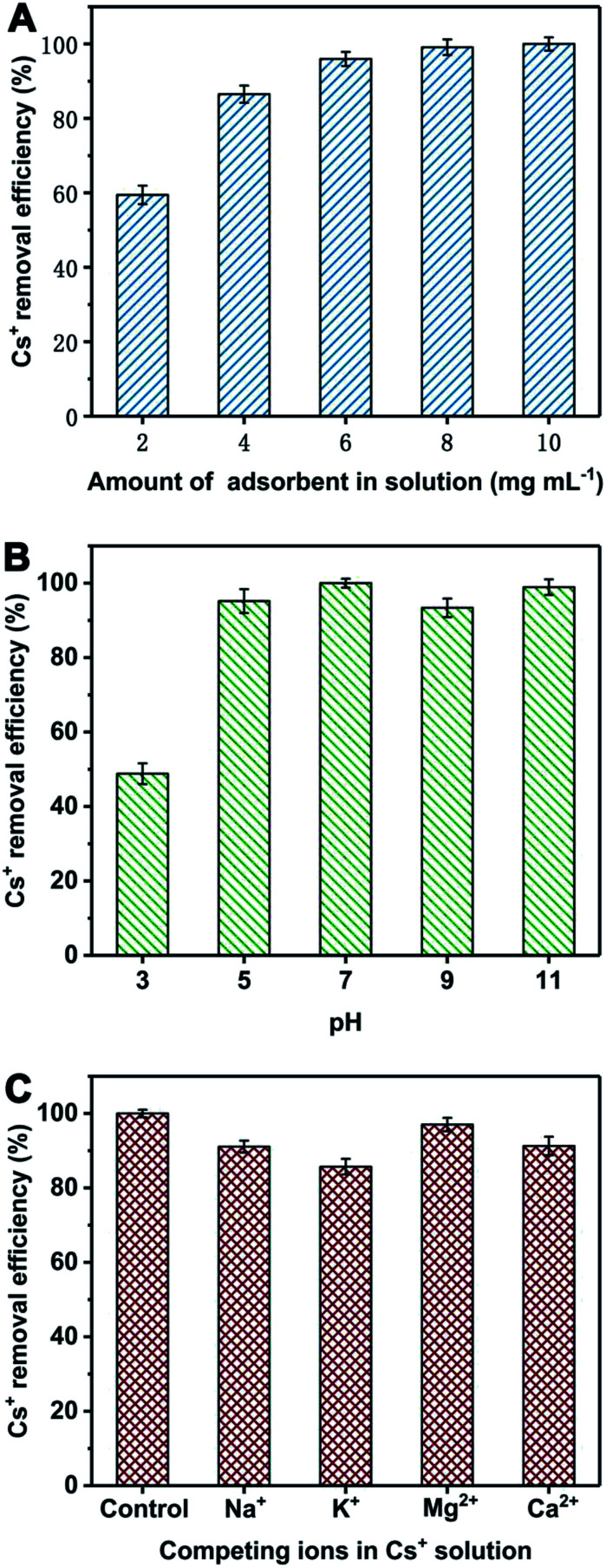
Cs^+^ removal efficiency of PB@PUS-2 sample under different adsorbent amount (A), pH (B) and competing cations (C). Data represent “mean ± SD” from three independent experiments.

The Cs^+^ removal efficiency is related to the amount of adsorbent in the solution. Thus, they were tested under different adsorbent amount and given in Fig. 6A. The Cs^+^ removal efficiency is obviously raised as the increase of adsorbent amount. When adsorbent amount is 6 mg mL^−1^, the Cs^+^ removal efficiency can reach 96%. When the amount of adsorbent is 8 mg mL^−1^, the Cs^+^ removal efficiency is more than 99%. The pH of solution is considered as an important factor to affect the Cs^+^ adsorption performances. Herein, the Cs^+^ removal efficiency of PB@PUS-2 at different pH was tested and shown in Fig. 6B. When pH is 7the Cs^+^ removal efficiency is the highest and reaches 99.9%. The Cs^+^ removal efficiency of PB@PUS-2 at pH 5–11 is more than 93%, which verifies that it can be applied in a wide range of pH values. The effect of common co-existing ions in seawater (K^+^, Na^+^, Ca^2+^ and Mg^2+^) was investigated and shown in Fig. 6C. The results reveal that co-existing metal ions can affect the adsorption capacity of PB@PUS-2 to some extent. The Cs^+^ removal efficiencies in the presence of Mg^2+^, Ca^2+^, Na^+^ and K^+^ are 97.0%, 91.3%, 91.1% and 85.7%, respectively. The influence of competing cations may be related to the ionic radius, hydrated ionic radius, charge-radius and electronegativity. In general, PB@PUS-2 sample has highly selective adsorption ability for Cs^+^. As is shown in the inset of Fig. 5, PB-induced blue colour and adsorbent fragments-induced murky did not appear during the sorption process. After adsorption, adsorbents were removed from the solution. The remaining solution was near colourless and transparent. There was no obvious suspended matter and precipitation. Neither PB particles nor PU fragments was released from bulk sponge during adsorption and separation processes, which indicate that PB/PU sponge has good stability.

### 3.3 Dynamic adsorption properties

**Fig. 7:**
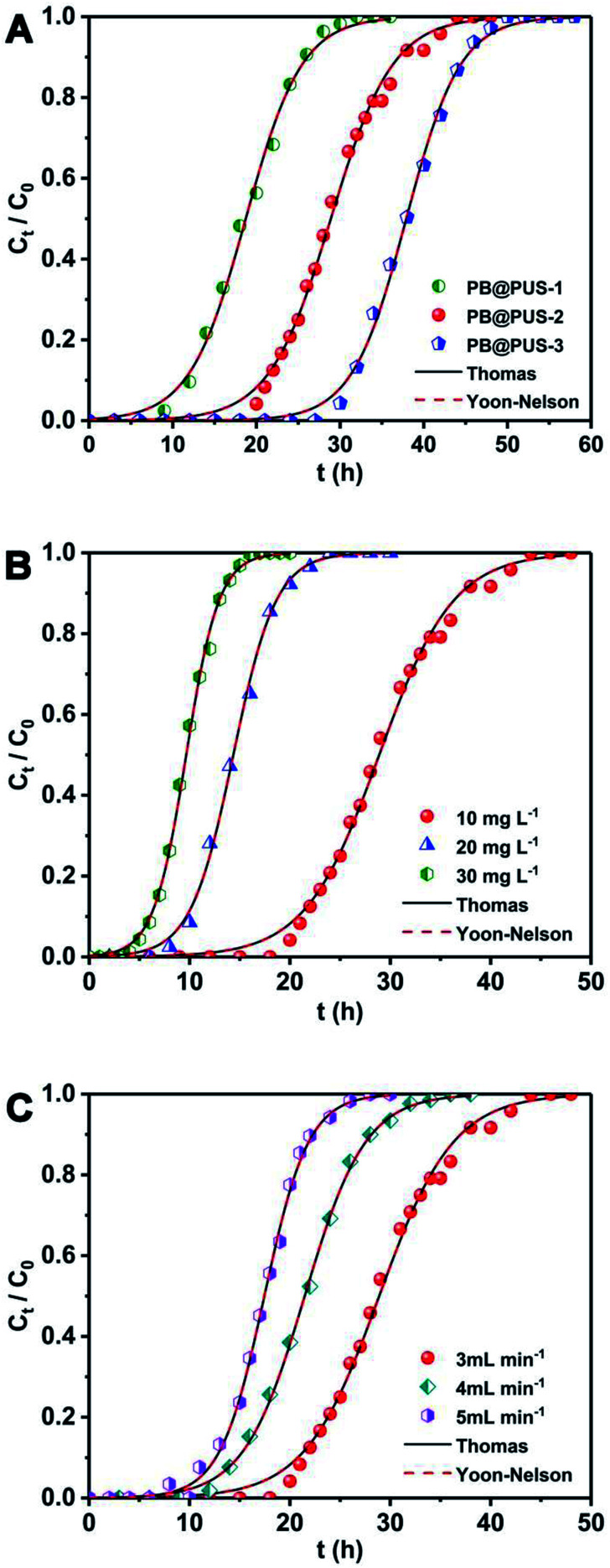
The Cs^+^ adsorption breakthrough curves of PB/PU sponges under different conditions. (A) Different adsorbent fabricated under different condition (initial Cs^+^ concentration: 10 mg L^−1^, flow rate: 3 mL min^−1^, bed height: 10 cm); (B) effect of initial Cs^+^ concentration (flow rate: 3 mL min^−1^, bed height: 10 cm, adsorbent: PB@PUS-2); (C) effect of flow rate (bed height: 10 cm, initial Cs^+^ concentration: 10 mg L^−1^, adsorbent: PB@PUS-2).

Fixed-bed column experiments under different conditions were carried to investigate the effect of adsorbent, initial Cs^+^ concentration and flow rate of solution. The breakthrough curves were obtained and shown in Fig. 7. The adsorption data are summarized in Table 3. As is shown in Fig. 7A, different adsorption behaviours for Cs^+^ in the fixed-bed column were observed in samples which were prepared under different concentration of precursor solution. Among three samples, PB@PUS-1 was the first one to reach the penetration point and depletion point, which reveals that the adsorption capacity of PB@PUS-1 was the lowest. PB@PUS-3 exhibits the best adsorption property. This is mainly related to the size and load of PB nanoparticles on sponge surface. The content of PB is higher in the sample which was prepared using the precursor solution of high concentration. As is shown in Fig. 7B, the breakthrough curve is smoother and the breakthrough rate is slower when the initial Cs^+^ concentration is lower. Both the breakthrough time and saturation time of the column decrease with the increase of initial Cs^+^ concentration. An increased initial cesium concentration leads to the steeper slope of breakthrough curves because of the faster mass-transfer flux from the bulk solution to the particle surface due to the increased diffusion coefficient. Higher initial cesium concentration results in better column performance with the increase of driving force and decrease in the adsorption zone length for adsorption process.^22^ The flow rate plays an important role in the fixed-bed column adsorption process. As is shown in Fig. 7C, the breakthrough curves are steeper at the higher flow rate. Both the breakthrough time and saturation time of the column obviously decrease with the increase of flow rate. The breakthrough time decreases from 18 h to 9.4 h. Similarly, the saturation time decreases from 47 h to 27 h. In addition, the breakthrough adsorption capacity, saturation adsorption capacity and removal efficiency all slightly decrease with the increase of flow rate. This can be attributed to the fact that the increased flow rate reduces the residence time of Cs^+^ in the column, which makes Cs^+^ do not have enough time and opportunity to contact with the binding sites of PB@PUS. At higher flow rate, there is not enough time for adsorption equilibrium to be reached.

**Table 3:** Parameters of the fixed bed column for Cs^+^ removal by PB/PU sponges under different conditions^a^

Adsorbent	Experimental conditions			Experimental parameters of breakthrough curves					
	*C*_0_ (mg L^−1^)	*v* (mL min^−1^)	*H* (cm)	*t*_b_ (h)	*t*_s_ (h)	*V*_eff_ (mL)	*q*_b_ (mg g^−1^)	*q*_s_ (mg g^−1^)	*R*_total_ (%)
PB@PUS-1	10	3	10	9	32	5760	15.4	32.2	55.9
PB@PUS-2	10	3	10	18	47	8460	30.8	51.8	61.2
PB@PUS-3	10	3	10	28.5	52	9360	48.6	67.0	71.6
PB@PUS-2	20	3	10	8	24	4320	27.4	49.2	57.0
PB@PUS-2	30	3	10	5	17	3060	25.1	50.6	55.2
PB@PUS-2	10	4	10	12.5	35	8400	28.5	50.3	59.9
PB@PUS-2	10	5	10	9.5	27	8100	26.8	48.9	58.1

a *C*_0_ = influent concentration (mg L^−1^), *v* = flow rate (mL min^−1^), *H* = bed height (cm), *t*_b_ = breakthrough time (h), *t*_s_ = saturation time (h), *V*_eff_ = effluent volume (mL), *q*_b_ = adsorption at breakthrough (mg g^−1^), *q*_s_ = adsorption at saturation (mg g^−1^), *R*_total_ = total cesium removal at saturation (%).

The breakthrough curves can be used to predict the efficiency of fixed-bed column system. Therefore, breakthrough curves under different conditions were analyzed and fitted using the Thomas model and Yoon–Nelson mode.^38^ The Thomas model is one of the most widely used breakthrough models for predicting breakthrough curves, which assumes Langmuir kinetics for adsorption and desorption with no axial dispersion. The Yoon–Nelson model is based on the assumption that the decrease in the probability of each adsorbate to be adsorbed is proportional to the probability of its adsorption and breakthrough on the adsorbent.^39^ The Thomas model and Yoon–Nelson model can be expressed as the eqn (12) and (13), respectively.12
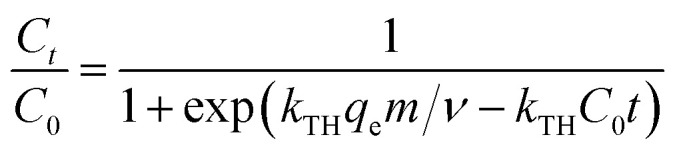
13
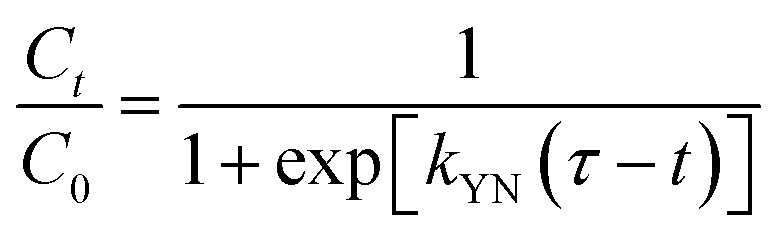
where *C*_0_ (mg L^−1^) is the inlet cesium concentrations, *C*_*t*_ (mg L^−1^) is the outlet cesium concentrations at time *t*, *k*_TH_ (mL min^−1^ mg^−1^) is the Thomas rate constant, *q*_e_ (mg g^−1^) is the adsorption capacity, *m* (g) is the amount of adsorbent in the column, *v* (mL min^−1^) is the volumetric flow rate, *k*_YN_ (h^−1^) is the Yoon–Nelson rate constant that depends on the diffusion characteristics of the mass transfer zone, and *τ* (h) is the time required for 50% adsorbate breakthrough.

The curves and parameters fitting with Thomas and Yoon–Nelson models are given in Fig. 7 and Table 4. The correlation coefficients of Thomas and Yoon–Nelson models are high, which indicate that they are able to describe the dynamic behaviour in the fixed-bed column. As the initial cesium concentration increases, the value of *k*_TH_ decreases because the driving force for adsorption is the concentration difference of cesium ions between adsorbents and solution. The value of *k*_TH_ increases with the increase of the flow rate. According to the Thomas model, the theoretical saturated adsorption capacities of three samples are 33.7 mg g^−1^ (PB@PUS-1), 52.0 mg g^−1^ (PB@PUS-2) and 68.2 mg g^−1^ (PB@PUS-3), respectively. They are consistent with the results of static adsorption experiments.

**Table 4:** Parameters of the Thomas and Yoon–Nelson models under different conditions

Adsorbent	*C*_0_ (mg L^−1^)	*v* (mL min^−1^)	*H* (cm)	Thomas model		Yoon–Nelson model		
				*k*_TH_ (mL mg^−1^ min^−1^)	*q*_e_ (mg g^−1^)	*k*_YN_ (h^−1^)	*τ* (h)	*R* ^2^
PB@PUS-1	10	3	10	0.496	33.7	0.298	18.7	0.997
PB@PUS-2	10	3	10	0.451	52.0	0.271	28.9	0.996
PB@PUS-3	10	3	10	0.514	68.2	0.308	37.9	0.998
PB@PUS-2	20	3	10	0.391	51.7	0.469	14.4	0.998
PB@PUS-2	30	3	10	0.341	52.2	0.613	9.66	0.998
PB@PUS-2	10	4	10	0.550	51.5	0.331	21.4	0.999
PB@PUS-2	10	5	10	0.757	52.4	0.454	17.5	0.998

### 3.4 Comparison of adsorption capacities with various adsorbents

As is shown in Table 5, the Cs^+^ adsorption capacity of as prepared PB/polyurethane sponges is comparable with other available adsorbents in previous publications. This kind of adsorbent has the following advantages: (1) they have good stability and are not easy to be released from the bulk adsorbent, which avoids the secondary contamination; (2) they have obvious porous structures and can ensure the smooth flow of solution among adsorbents, which can be applied in fixed-bed columns; (3) they have good compressibility, which can be conveniently stored, efficiently used and easily post-treated in wastewater treatment. This fabrication strategy and method can be easily applied to prepare other similar porous hybrid adsorbents.

**Table 5:** Comparison of adsorption capacities for Cs^+^ onto various adsorbents

Adsorbents	*Q*_m_ (mg g^−1^)	References
PB/Fe_3_O_4_/GO	55.56	15
PB/magnetic nanoclusters	45.87	18
CsTreat (K_2_CoFe(CN)_6_)	32.36	21
PB/cellulose nanofibers	139	24
KCuHCF/Fe_3_O_4_/PVA hydrogel	82.8	25
PB/RGO foam	18.67	28
PB/magnetic hydrogel beads	41.15	32
PB@PUS-3 foam	68.6	This study

## 4. Conclusions

In this study, uniform and well-dispersed Prussian blue nanoparticles were successfully prepared on the porous skeleton of sponge *via* an *in situ* radiation chemical route. Batch and fixed-bed column experiments were carried out to investigate their adsorption performances for cesium ions. PB@PUS exhibits good selective adsorption property for cesium ions in a wide range of pH, whose maximal adsorption capacity and removal efficiency reached 68.6 mg g^−1^ and 99%, respectively. The adsorption processes can be described by Langmuir isotherm adsorption model and pseudo-second-order adsorption kinetic model. The breakthrough time and exhaustion time of column are closely related to the PB content in PB@PUS, flow rate and initial cesium ions concentration. The breakthrough curves are well fitted by the Thomas model and Yoon–Nelson model. In addition, the PB@PUS is light, stable and compressible, which is good for use and storage. The as prepared samples can be applied as adsorbent to efficiently remove cesium ions from radioactive wastewater.

## Conflicts of interest

There are no conflicts to declare.

## Supplementary Material
